# Impact of hypoxia on alveolar bone dynamics and remodeling

**DOI:** 10.1016/j.heliyon.2024.e40868

**Published:** 2024-12-02

**Authors:** Sangeetha Narasimhan, Sausan Al Kawas, Shishir Ram Shetty, Hiba Saad Al-Daghestani, Rani Samsudin

**Affiliations:** Department of Oral and Craniofacial Health Sciences, College of Dental Medicine, University of Sharjah, United Arab Emirates

**Keywords:** Hypoxia, Alveolar bone, Socket healing, HIF

## Abstract

Oxygen is a fundamental requirement for cellular metabolism. Hypoxia is a state of oxygen deprivation of the tissues. Cells develop numerous adaptive mechanisms to survive hypoxic insult. Alveolar bone is a unique structure that encases and protects the tooth. Literature reports that hypoxia, in all forms, impacts alveolar bone health. The hypoxia-inducible pathway appears to play a key role in mediating changes in alveolar bone metabolism. Embryonic hypoxia plays a vital role in craniofacial skeletal development. Further, hypoxia has been anticipated in the repair of extraction sockets. Alveolar bone cells respond distinctly to hypoxic conditions with both beneficial and detrimental effects. Studies have demonstrated enhanced alveolar bone resorption upon hypoxic stimuli. However, hypoxia has also been shown to have potential therapeutic effects on alveolar bone by triggering an angiogenic response. Additionally, the type, duration, and mode of hypoxia are critical in triggering varied responses in alveolar bone metabolism. The main objective of this review is to recapitulate the effects of different types of hypoxia on the tooth supporting apparatus and to analyze some of the presumptive mechanisms underlying hypoxia-induced changes in alveolar bone remodeling.

## Abbreviations

ANGPTL4: Angiopoietin-like 4
BMD: Bone Mineral Density
BMPs: Bone Morphogenetic Proteins
BMSCs: Bone Marrow Mesenchymal Stem Cells
CCH: Chronic Continuous Hypoxia
CIH: Chronic Intermittent Hypoxia
CoCl2: Cobalt Chloride
Cyp1a1: Cytochrome P450 1A1
DMOG: Dimethyloxalylglycine
ERS: Endoplamic Reticulum Stress
FGFs: Fibroblast Growth Factors
FOXO1: Forkhead Box Protein O1
HIF-1α: Hypoxia Inducible Factor 1α
HIFs: Hypoxia Inducible Factors
HMA: Hypoxia Mimetic Agents
hPDLSCs: Human Periodontal Ligament Stem Cells
IL-6: Interleukin 6
MCSF: Macrophage Colony Stimulating Factor
mRNA: Messenger RNA
NF-κB: Nuclear factorκB
OPG: Osteoprotegerin
PDGFs: Platelet-Derived Growth Factors
PDL: Periodontal Ligament
PGE2: Prostaglandin E2
PHD: Prolyl Hydroxylase
pO2: Partial pressure of Oxygen
RANK: Receptor activator of nuclear factor kappaB
RANKL: Receptor activator of nuclear factor kappaB ligand
ROS: Reactive Oxygen Species
SD Rats: Sprague-Dawley Rats
TNFα: Tumor Necrosis Factor α
VEGF: Vascular Endothelial Growth Factor
VHL: von Hippel-Lindau
WR: Wistar Rats

## 1 Introduction

Oxygen is essential for normal cellular physiology. Hypoxia is a state of reduced tissue oxygen concentration [1]. Cells sense and respond to hypoxia by developing numerous adaptive mechanisms to facilitate survival. This process is mainly regulated by hypoxia - inducible factors (HIFs) [2]. Hypoxia can occur physiologically due to environmental factors or pathologically in the context of disease, both of which significantly impact bone health [3]. While embryonic hypoxia supports skeletal development, neonatal hypoxic exposure can disrupt bone remodeling processes during the early postnatal weeks. Even a brief hypoxic exposure during growth can lead to defects in jaw size and bone quality [4]. Additionally, hypoxia during puberty has been shown to impair maxillofacial bone growth in rats [5].

The alveolar bone is a unique osseous structure that surrounds and protects the tooth. Alveolar bone remodeling is complex and is influenced by occlusal forces, oral biofilms, and the periodontal ligament (PDL) status. Bone cells need an adequate supply of oxygen to sustain their cellular and physiological functions [6]. Clinical research has shown that hypoxia of the periodontium greatly affects alveolar bone remodeling, bone mineral density (BMD), and socket healing [[7], [8], [9]]. Various pathways have been proposed and studied to understand how hypoxia affects alveolar bone remodeling. Data from previous reports indicate that hypoxia can significantly affect both osteoblast and osteoclast differentiation and thereby modulate both alveolar bone formation and resorption, with its effects heavily dependent on the type and duration of hypoxic exposure [3]. Chronic or prolonged hypoxia often results in alveolar bone loss, driven by hypoxia inducible factor 1α (HIF-1α) - mediated inflammatory pathways that control osteoclastogenesis [7,10,11]. Conversely, short-term intermittent hypoxia in vivo has been shown to stimulate alveolar bone formation by promoting osteogenic differentiation, primarily through the activation of the HIF-1α - controlled genes that enhance angiogenesis and osteoblast activity [9]. Moreover, hypoxia impacts alveolar bone remodeling through various mechanisms depending on the stimuli. How different hypoxic factors activate distinct pathways in bone metabolism remains unclear [3]. Therefore, it is essential to recognize the underlying pathogenesis of alveolar bone remodeling during various hypoxic exposures to effectively harness these mechanisms for therapeutic purposes. This review presents a summary of the potential mechanisms by which hypoxia influences alveolar bone metabolism, emphasizing how the type and duration of hypoxia could be critical factors in driving changes in the health of the periodontium.

## 2 Alveolar bone: Structure and function

**Fig. 1:**
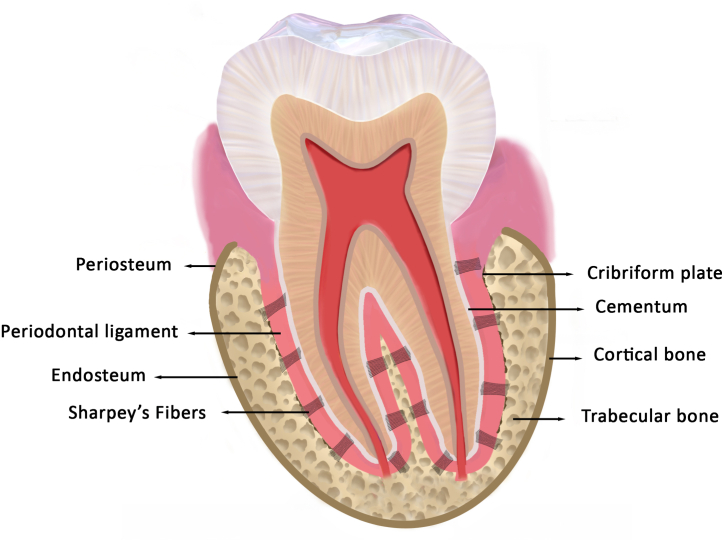
Schematic representation of the structure of Alveolar Bone.

The tooth-bearing processes of the jaws are known as the alveolar process or alveolar bone. Originating from the neural crest-derived ectomesenchyme, the alveolar process develops through intramembranous ossification during the late bell stage of odontogenesis. Alveolar processes are considered as tooth - dependent osseous structures that develop alongside tooth formation and disappear after tooth loss [12]. Alveolar bone is a mineralized connective tissue composed of 23 % mineralized tissue, 37 % organic matrix, and 40 % water [13]. It comprises the alveolar bone proper (also called bundle bone, cribriform plate, or lamina dura) and the supporting alveolar bone, which includes cortical and trabecular bone. The alveolar bone proper, spanning 0.1–0.4 mm in thickness, is the innermost component lining the tooth socket that houses the Sharpey's fibers from the PDL. The buccal and lingual cortical plates encase the alveolar bone proper on the outer surface, merging at the alveolar bone crest. The supporting trabecular bone lies between the alveolar bone proper and the cortical plates. The periosteum and endosteum line the outer and inner surfaces of the cortical plates, respectively, while the PDL lines the innermost surface of the alveolar bone proper [Fig. 1] [6]. The alveolar bone and PDL are closely interconnected in both development and function. They actively undergo remodeling in both homeostasis and disease [13]. The PDL cells and their secretory products regulate alveolar bone formation and resorption [14]. The principal functions of the alveolar bone include protection of tooth roots and masticatory support. Additionally, the medullary spaces in the alveolar bone serve as a potential source of hematopoietic and mesenchymal stem cells and act as a calcium reservoir [6].

## 3 Alveolar bone remodeling

Bone remodeling is a cyclic process that replaces old bone with new, ensuring the preservation of its mechanical and metabolic roles [15]. Approximately 10 % of the adult skeleton undergoes turnover every year. The masticatory forces induce 2–4 times greater mechanical load on the alveolar bone complex compared to the extraoral skeleton, leading to a 3–6 times higher remodeling rate [6]. Alveolar bone remodeling is chiefly mediated by the PDL cells [8]. Osteoblasts are specialized cells of mesenchymal origin that secrete bone matrix and coordinate skeletal mineralization [16]. Osteoclasts are large, multinucleated cells of hematopoietic (monocyte/macrophage) lineage that regulate bone resorption. Maintaining bone homeostasis requires a balance between osteoclast-mediated bone resorption and osteoblastic bone formation [12]. The Wnt and osteoprotegerin/Receptor activator of nuclear factor kappaB ligand/Receptor activator of nuclear factor kappaB (OPG/RANKL/RANK) pathways largely control the alveolar bone remodeling in health and disease [17,18]. RANKL and OPG are crucial in regulating alveolar bone remodeling. RANKL primarily drives osteoclastogenesis, while OPG acts as a soluble decoy receptor, inhibiting this process by preventing RANKL from binding to its receptor, RANK. The balance between RANKL and OPG expression plays a significant role in bone remodeling: a higher RANKL/OPG ratio tends to promote bone resorption, whereas a lower ratio favors bone formation [18].

## 4 Factors influencing alveolar bone remodeling

The alveolar bone is a dynamic structure that experiences ongoing cycles of bone resorption and new bone formation [19]. The integration of the alveolar bone with the PDL, dentition, and its proximity to oral biofilms marks it as a distinctive type of osseous tissue. As such, alveolar bone remodeling is significantly influenced by occlusal forces, periodontal health, and oral microbiota [13,20,21]. The PDL is a specialized connective tissue that connects the tooth to the alveolar bone and serves as a potential source of bone remodeling progenitor cells. Masticatory forces generated by the dentition are transmitted to the alveolar bone through the PDL [8]. Increased mechanical forces lead to increased bone formation and decreased resorption, while reduced occlusal forces result in increased bone resorption. This can be explained by the fact that the loss of occlusal forces, as in the case of tooth extraction, and the consequent loss of the PDL, leads to the complete resorption of the alveolar process [13]. Previous periodontal research has shown that commensal oral flora plays a crucial role in maintaining alveolar bone homeostasis within a healthy periodontium. The highly permeable dentogingival junctional epithelium allows microbes from the dental biofilm to mediate immune responses, which induces paracrine signaling and directly modulates alveolar bone remodeling [6]. However, when the ecological balance of the oral microbiota is disrupted, it can lead to alveolar bone resorption [21]. Notably, studies have demonstrated that alveolar bone loss is significantly reduced in germ-free mice, highlighting the impact of the oral microbes on bone health [22,23].

## 5 Hypoxia: Definition, types, and causes

Oxygen is a crucial component of aerobic metabolism. The state of reduced oxygen concentration and partial pressure within the mammalian cells is termed Hypoxia. American physiologist Carl Wiggers first coined the term hypoxia in 1941 from a Greek word hypo-meaning under/below and Latin oxygenium meaning oxygen [24]. Tissue hypoxia manifests in two forms: short-term (as in ischemia) or long-term, (lasting several hours to days), such as when staying at high altitudes [25]. Short-term hypoxia can be continuous or intermittent [9,26]. Chronic hypoxia can either be continuous or intermittent-where oxygen levels fluctuate between low and normal. About 2 % of the world's population living in high altitude are exposed to chronic continuous hypoxia (CCH). Sleep-disordered breathing, like obstructive or central sleep apnea, is an example of chronic intermittent hypoxia (CIH) [11]. Additionally, based on the varying levels of intensity, hypoxia can be mild, moderate, or severe [27].

The three primary causes of tissue hypoxia include: hypoxemia, impaired oxygen delivery to tissues, and diminished tissue oxygen utilization [28]. In humans, hypoxia can result from diseases, climate change, or high-altitude living [24]. Hypoxic stress can lead to significant cellular adaptations or result in cell death, depending on the cell type, its ability to adapt to hypoxic conditions and its metabolic requirements [28].

## 6 Role of hypoxia-inducible factors (HIFs)

**Fig. 2:**
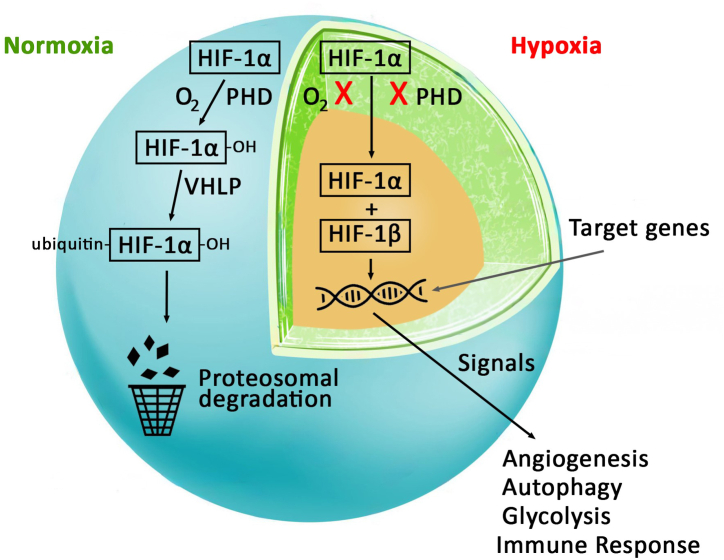
Schematic representation of HIF-1α in Normoxia and Hypoxia.

HIFs are a family of transcription factors that are key regulators of oxygen homeostasis. HIFs comprise an oxygen-sensitive HIF-α (HIF-1α, HIF-2α, HIF-3α) and oxygen-independent HIF-β subunits. While HIF-1α is ubiquitously expressed across all body tissues, HIF-2α and HIF-3α are found only in a few specific tissues. In normoxia, HIF-α is hydroxylated by oxygen dependent prolyl hydroxylase (PHD) leading to the recruitment of von Hippel-Lindau (VHL) protein that promotes proteasomal degradation of HIF-α. However, under hypoxic conditions, prolyl hydroxylase is inhibited and HIF-α becomes stable and accumulates within the cell. Further, HIF-1α interacts with HIF-β to form a dimer that translocates to the nucleus and activates gene transcription factors which enables the cells to adapt and survive in a hypoxic environment [29]. Multiple genes that control cellular metabolism, angiogenesis, glycolysis, immune response, autophagy, cell growth, and proliferation, are potential targets of HIFs [Fig. 2] [30]. In addition, HIF-1α has been implicated in regulating different bone-related activities, such as bone development, normal remodeling, disease-related damage, and healing process [31]. The HIF-mediated bone metabolism is undeniably complex, requiring careful comparison, particularly when considering different physiological or pathological contexts [3].

## 7 Hypoxia of the periodontium

Hypoxia in the oral tissues can either be due to various dental pathologies and their treatments that cause local trauma, inflammation, or ischemia. The periodontium is a complex of specialized tissues that facilitates tooth anchorage and proprioception through a desmodontal attachment [32]. The oxygen concentration of the normal periodontium ranges from 2.9 % to 5.7 % [33]. Periodontitis is the inflammatory disease of the PDL that causes resorption of alveolar bone and the reduction of BMD [34]. Hypoxia is considered to influence the development and progression of periodontitis [32]. Compromised local blood circulation in periodontitis further intensifies the hypoxic microenvironment [33]. Moreover, tissues affected by periodontitis show a considerable influx of inflammatory cells, which increases oxygen consumption in the area [35]. Studies have demonstrated a reduced partial pressure of oxygen (pO2) in periodontitis compared to a healthy periodontium. Lower oxygen concentration in periodontitis is ideal for the growth of gram-negative bacteria whose metabolites deepen PDL hypoxia. The oxygen tension at the bottom of periodontal pockets is estimated to be just 13.3 mmHg in untreated periodontitis patients [36]. Smoking can significantly reduce the oxygen content in periodontal tissues, with smokers having only two-thirds of the oxygen levels found in non-smokers [37]. Hitoshi M et al. reveal that trauma caused by excessive occlusal forces could result in oxygen deficiency in the periodontium [38]. Further, compressive forces generated by orthodontic treatment cause hypoxia of alveolar bone and PDL by reducing the blood flow [33]. Sleep-related breathing disorders such as obstructive sleep apnea can also lead to hypoxia of oral tissues [5].

## 8 In vitro and in vivo hypoxia induced alveolar bone models

The impact of hypoxia on alveolar bone has been widely investigated in recent years using both in vitro and in vivo animal models [Table 1]. Sprague-Dawley (SD) rats and Wistar rats (WR) were the most used animals for in vivo hypoxic studies, followed by Wild-type C57BL/6J mice [8,10,31]. In vitro experiments employed human periodontal ligament stem cells (hPDLSCs), mouse macrophage cell lines, and bone marrow mesenchymal stem cells (BMSCs) [[26], [34], [36]]. For the animal experiments, hypoxia was induced through methods such as unilateral nasal obstruction or hypoxic pressure chambers [7,34]. The cell lines were cultured under low oxygen tension or subjected to chemical mode of hypoxia using cobalt chloride (CoCl2) [26]. Across the studies, there was a consistent increase in HIF-1α expression. The primary outcomes of most of these studies suggest that hypoxia in various modes and duration leads to alveolar bone loss. Oxygen deprivation in periodontal tissues was found to accelerate the progression of periodontitis [39]. CCH generally resulted in cortical bone loss and was linked to osteoclastogenesis, increased autophagy, and decreased osteogenic differentiation, while CIH further exacerbated bone loss in the cortical and interradicular areas of the alveolar bone [7,10,11,34]. Studies also reported a hypoxia-induced reduction in BMD and alveolar bone quality [34]. On the contrary, a few studies showed an increased BMD in alveolar bone, reduced periapical bone loss, enhanced osteoblastic differentiation, and accelerated socket healing with induced hypoxia [9,26,31].

**Table 1:** In vivo & In vitro studies evaluating the effect of hypoxia on alveolar bone.

Study	Model	Study type	Hypoxia type & Duration	Hypoxia Mode		Outcome
Terrizzi AR et al., 2013 [39]	Wistar rats	In vivo	CIH- 18 h/day for 3months	Hypoxia chamber	↑TNF-α, PGE2	↑EP, alveolar bone loss
Yu XJ et al., 2015 [18]	HPLCs	In vitro	Short term continuous hypoxia	2 % O2 culture	↑HIF-α, RANKL/OPG ratio	↑ osteoclastogenesis
Terrizzi AR et al., 2016 [10]	Wistar rats	In vivo	CCH - 23.5 h/day for 3months	Hypoxia chamber	↑iNOS, TBA-RS & ROS	↑alveolar bone loss
					↓TNF-α	
Oishi S et al., 2016 [8]	SD rats	In vivo	CCH - 8 h/day/3weeks	Hypoxia chamber	↑HIF-1α, VEGF, ALP, BMP-2	↑BMD of alveolar bone
Terrizzi AR et al., 2018 [11]	Wistar rats	In vivo	CCH - 24 h/day for 3months	Hypoxia chamber	↑HIF-1α, PGE_2_, CTX-I	↑cortical bone loss in both groups
			CIH -18 h/5 day/week for 3months		↓AQP-5	Additional Interradicular bone loss in intermittent hypoxia group
Hirai K et al., 2018 [31]	Wild-type C57BL/6J mice	In vivo	CCH	HIF-1 activation using Adenoviral Vector System	↓IL-1α, TNFα, NF-κB	↓Periapical bone loss
Terrizzi AR et al., 2021 [7]	Wistar rats	In vivo	CCH-24 h/day for 3months	Hypoxia chamber	↑PGE2	↑alveolar cortical bone loss
			CIH -18 h/5day/week for 3months			
Kim JE et al., 2022 [40]	Wistar rats	In vivo	CCH - 6 weeks	Unilateral nasal obstruction	↑HIF-1α, RANKL, IL-6, TNFα, RAGE	↑alveolar bone loss
					↓OPG, BMP-2, BMP-7	↓alveolar bone quality
Xu Y et al., 2023 [41]	SD rats	In vivo	CCH - 6 weeks	Unilateral nasal obstruction	↑LC3, ↓ P62	↓osteogenic differentiation
	BMSCs	In vitro	Short term continuous hypoxia 6 h s & 12 h	5 % O2 culture	↓p62, p-mTOR/mTOR, RUNX2, BSP, OCN, SOX9, Osterix & COL2 ↑ LC3, Beclin 1, HDAC6, HIF-α and FOXO1 ↑HIF-1α, RANKL/OPG Ratio	↑autophagy ↓ osteogenic differentiation
Xu Y et al., 2023 [34]	Wistar rats	In vivo	CCH -5 weeks	Unilateral nasal obstruction	↓Cyp1a1 gene	↓BMD of alveolar bone
	BMSCs	In vitro	Short term continuous hypoxia/3 days	5 % O2 culture	↑HIF-1α, RANKL, OPG ↓Cyp1a1	↓ reduced osteogenic differentiation
Qi X et al., 2023 [36]	HIF-1α CKO mice	In vivo	CCH		↑HIF-1α ANGPTL4	↑alveolar bone loss
	RAW264.7 mouse macrophage cell line	In vitro	Short term continuous hypoxia/2 days	CoCl2 in cell culture	↑HIF-1α, ANGPTL4	↑osteoclastogenesis
Linawati L et al., 2023 [9]	SD rats	In vivo	Short term Intermittent hypoxia 30min/day for 1,3,5, 7days	Hypobaric chamber	↑HIF‐1α & VEGF	↑extraction socket healing
Fan Z et al., 2024 [26]	SD rats	In vivo	CCH - 28 days	Intraperitoneal injection of CoCl2	↑HIF‐1α, RUNX2, OCN	↑alveolar bone defect repair
	hPDLSCs	In vitro	Short term continuous hypoxia/2 days	Incubation with CoCl2	↑RUNX2, OCN, Col1A1, and ALP	↑osteogenic differentiation

**Abbreviations::** ↑: Increase; ↓: decrease or reduced; TNF-α: tumor necrosis factor α; PGE2: prostaglandin E2; EP: Experimental periodontitis; HPLCs: human periodontal ligament cell; HIF: Hypoxia inducible factor; RANKL: Receptor activator of nuclear factor kappaB ligand; OPG: Osteoprotegerin; iNOS: Inducible nitric oxide synthases; TBA-RS: Thiobarbituric acid reactive species; ROS: Reactive oxygen species; SD: Sprague-Dawley; VEGF: Vascular endothelial growth factor; ALP: alkaline phosphatase; BMP-2: Bone morphogenic protein 2; BMD: Bone mineral density; CTX-I: C-Terminal Telopeptide of Collagen Type I; AQP-5: Aquaporin 5; IL-1α: Interleukin 1 alpha; NF-κB: Nuclear factor-κB; IL-6: Interleukin 6; RAGE: Receptor for advanced glycation end products; BMP-7: Bone morphogenic protein 7; HDAC6: Histone deacetylase 6; FOXO1:Fork-head box protein O1; CoCl2: Cobalt chromium; BMSCs: Bone marrow stem cells; Cyp1a1: Cytochrome P450 1A1; ANGPTL4:Angiopoietin-like Protein 4; OCN: Osteocalcin; Col1A1: collagen type I alpha 1 chain; ALP: Alkaline phosphatase.

## 9 Hypoxia-induced signaling pathways associated with alveolar bone remodeling

### 9.1 Hypoxia induced alveolar bone resorption

#### 9.1.1 Altered RANKL/OPG expression ratio

**Fig. 3:**
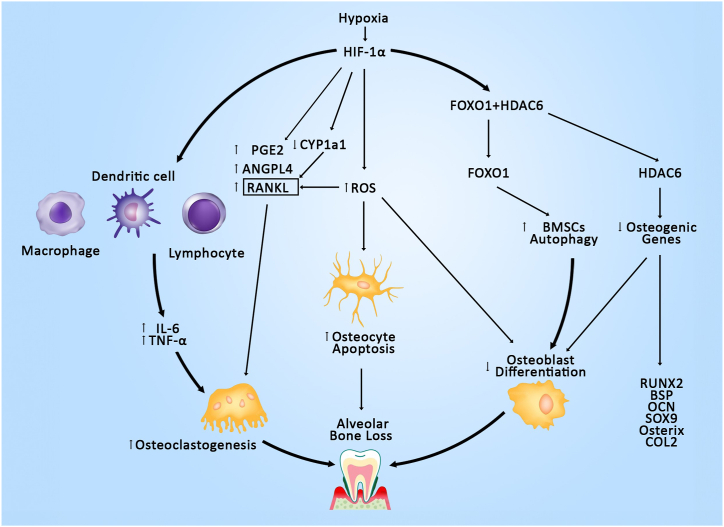
Hypoxia mediated pathways involved in Alveolar Bone Resorption.

Multiple pathways have been anticipated in the hypoxic-controlled alveolar bone remodeling [Fig. 3]. The key event in most of the hypoxia-induced alterations of bone is the activation of HIF-1α, which subsequently triggers several downstream mechanisms that influence bone metabolism and remodeling. RANKL-mediated osteoclastogenesis is a well-established factor in alveolar bone resorption. Yu XJ et al. demonstrated a significant increase in the RANKL mRNA and a decrease in OPG mRNA in the human periodontal ligament cells (HPLCs) subjected to short-term continuous hypoxia [18]. However, the exact mechanism by which HIF-1α activates RANKL remains unclear. Kim JE et al. demonstrated that HIF-1α activation increases inflammatory cytokines in hypoxic rats [40]. It can be hypothesized that HIF-1α may enhance RANKL overexpression by upregulating inflammatory cytokines. However, further research is necessary to validate this proposed pathway.

#### 9.1.2 The HIF-1α and ANGPTL4 pathway

Angiopoietin-like 4 (ANGPTL4) is a hypoxia-induced adipokine primarily known for regulating lipid metabolism and angiogenesis. Additionally, ANGPTL4 stimulates osteoclast activity and promotes bone resorption in vitro. Studies have indicated that HIF-1α significantly influences ANGPTL4 expression [42,43]. Chemically induced hypoxia in RAW264.7 mouse macrophage cell lines increased the expression of HIF-1α and ANGPTL4, leading to a higher number of osteoclasts, as evidenced by TRAP staining. Moreover, exogenous ANGPTL4 counteracted the inhibitory effects of HIF-1α siRNA on osteoclast differentiation by stimulating osteoclastogenesis. Therefore, under hypoxic conditions, HIF-1α facilitates ANGPTL4-mediated osteoclast differentiation in mouse macrophages [36].

#### 9.1.3 Reactive oxygen species (ROS) mediation bone destruction

Terrizzi et al. demonstrated that hypoxia-induced alveolar bone loss in rats is driven by elevated levels of ROS [10]. ROS are highly reactive, oxygen-containing molecules, and under hypoxic conditions, their levels can increase directly due to the activation of xanthine oxidase. An excessive accumulation of ROS can further stabilize HIF-1α in hypoxic tissues [25]. Elevated ROS levels act as secondary messengers in RANKL-mediated osteoclast differentiation, thereby enhancing bone resorption. Alongside the bone resorption, increased accumulation of ROS inhibits osteoblast differentiation and bone matrix mineralization [3]. The elevation of ROS in alveolar bone leads to osteocyte apoptosis by inducing endoplasmic reticulum stress (ERS) [44]. Given these factors, hypoxia-induced ROS generation plays a significant role in alveolar bone loss by impacting both bone formation and resorption pathways.

#### 9.1.4 Inflammation mediated bone destruction

Inflammation directly modulates bone remodeling and hypoxia is usually a feature of inflammation. HIF-1- mediated inflammatory pathway contributes to alveolar bone loss in animals subjected to hypoxia [7,39]. Prostaglandin E2 (PGE2) is the most abundant prostanoid in bone and is recognized as a potent promoter of bone resorption. It induces the expression of RANKL on osteoblastic cells through an autocrine/paracrine mechanism, which, in turn, promotes osteoclast differentiation from hematopoietic precursors [45]. Upregulation of HIF-1α in human PDL cells stimulated PGE2 production [7,11]. Elevated levels of PGE2 in hypoxic animals directly promoted alveolar bone loss through osteoclastogenesis. On the other hand, HIF-1α-mediated elevation of PGE2 and suppression of aquaporin 5 (AQP-5) indirectly modulate alveolar bone resorption by reducing salivary secretion [11]. Furthermore, HIF-1α activates macrophages, dendritic cells, and T and B lymphocytes, leading to the secretion of inflammatory cytokines such as TNF-α and IL-6 [40]. TNF-α is a key cytokine involved in inflammation and immunity, contributing to bone destruction by directly promoting osteoclast differentiation and maturation, as well as indirectly exposing the bone matrix [46]. IL-6 increases the number of osteoclasts by inhibiting apoptosis and enhancing their responsiveness to macrophage colony-stimulating factor (M-CSF) and RANKL [47]. In conclusion, hypoxia-driven inflammatory mediators contribute to alveolar bone destruction by enhancing osteoclastogenesis.

In contrast to studies that demonstrated hypoxia-driven inflammation leading to alveolar bone destruction, Hirai et al. showed a reduction in inflammation and periapical alveolar bone loss in HIF-1 activated mice. The activation of HIF-1 downregulated Receptor Activator of NF-*κB*, suppressed IL-1α, TNF-α, M1 macrophages, and osteoclastogenesis. Therefore, the HIF-mediated reduction in periapical bone loss appears to be due to the inhibition of osteoclasts rather than the promotion of osteoblasts and bone formation. Notably, this study did not induce hypoxia in the animals; instead, only HIF-1 was activated under normoxic conditions [31].

### 9.2 Hypoxia induced osteogenic pathways

#### 9.2.1 The HIF-1α and VEGF pathway

**Fig. 4:**
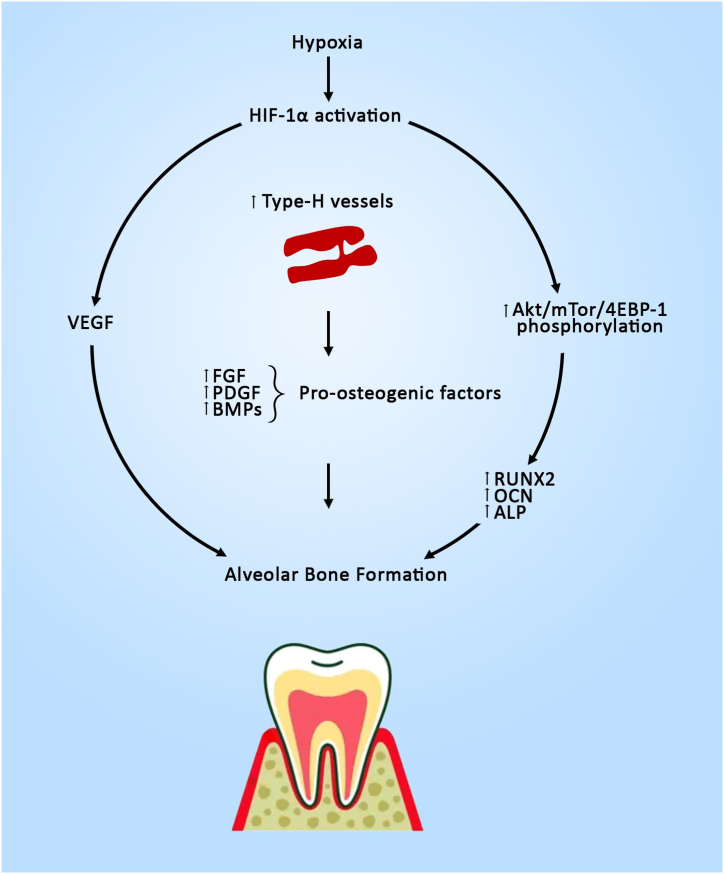
Hypoxia Mediated Pathways involved in Alveolar Bone Healing/Repair.

Vascular endothelial growth factor (VEGF) is one of the most important growth factors that regulate angiogenesis. It is a well-known fact that angiogenesis and osteogenesis are highly coupled [42]. Hypoxia appears to promote alveolar bone formation in growing rats by inducing the HIF-1α and VEGF pathways. The upregulation of HIF-1α under hypoxic conditions increased VEGF expression in osteoblasts [9]. Previous studies have revealed that osteoblast-derived VEGF affects bone development, repair, and regeneration by recruiting bone cell progenitors [42]. VEGF facilitates angiogenesis, promotes osteoblast differentiation, and migration in the PDL [8]. A similar pathway has been anticipated in acceleration of post-extraction socket healing in SD rats [Fig. 4] [9].

#### 9.2.2 The AKT/mTOR/4EBP‐1/HIF‐1α signaling pathway

Protein Kinase B (Akt) plays a vital role in maintaining alveolar bone physiology [48]. Research suggests that hypoxia is closely linked to Akt activation. A recent study revealed that short-term intermittent hypoxia elevated Akt/mTOR/4EBP-1 phosphorylation, which, in turn, promoted the osteogenic differentiation of hPDLSCs as evidenced by the increased expression of osteogenesis‐ related factors such as RUNX2, OCN, Col1A1, and ALP. Additionally, in vivo experiments demonstrated that hypoxia improved the repair of alveolar bone defects, underscoring the importance of the Akt/mTOR/4EBP-1/HIF-1α signaling pathway in this process [26].

#### 9.2.3 Suppression of osteogenic differentiation

Bone loss isn't solely attributed to resorption; it also involves reduced bone formation. Osteogenic differentiation is a pre-requisite for bone formation [49]. Evidence has demonstrated a correlation between autophagy activity and osteogenic differentiation. HDAC6 is a unique cytoplasmic deacetylase that promotes autophagy in cells and its upregulation has been reported in hypoxic mandibles [50]. Forkhead box protein O1 (FOXO1) is a transcription factor involved in autophagy regulation. Under normoxia, FOXO1 binds to HDAC6 in the cytoplasm. Recent reports show that hypoxia triggers autophagy by targeting FOXO1 [51]. Furthermore, hypoxia causes the dissociation of the FOXO1-HDAC6 complex, leading to their translocation into the nucleus. In the nucleus, FOXO1 activates autophagy-related genes p-ULK/ULK, Beclin 1 and LC3B, while HDAC6 deacetylates osteogenic genes and thereby lowers the levels of RUNX2, BSP, OCN, SOX9, Osterix and COL2. This study findings verify that hypoxia mediated dissociation of HDAC6-FOXO1 increased BMSCs autophagy and inhibited osteogenic differentiation thereby resulting in alveolar bone loss of rat mandibles [Fig. 3] [41].

Cytochrome P450 1A1 (Cyp1a1) is an extrahepatic enzyme that plays a role in oxidative metabolism. In addition, Cyp1a1 influences osteogenic metabolism and decreases bone resorption by inhibiting RANKL. Hypoxia-mediated HIF-1α activation suppressed osteogenic differentiation of bone marrow stem cells (BMSCs) by deactivating Cyp1a1 resulting in a reduction of the bone marrow density in the mandible [34].

## 10 Effect of hypoxia in alveolar socket healing

Tooth extraction is a routine dental procedure that compromises the alveolar bone's blood supply. Ischemia creates a hypoxic microenvironment in the bone, resulting in osteocyte apoptosis [52]. Apoptotic osteocytes block the Wnt/β-catenin pathway by secreting sclerostin, thereby preventing osteoblast differentiation. Furthermore, they release RANKL, which promotes osteoclastic differentiation and results in alveolar bone resorption, a common clinical observation following tooth extraction [53]. A cascade of biological events is initiated following tooth extraction to restore the alveolar socket. This process, known as socket healing, involves both the alveolar bone and surrounding soft tissues and begins immediately post-extraction, extending up to approximately 6 months. However, research indicates that the modeling and remodeling of alveolar bone persist for over a year following extraction [54].

Preservation of alveolar ridge dimensions is an essential prerequisite for removable dentures and implant placement [55]. Compared to normoxia, hypoxic conditioning has superior therapeutic potential in inducing bone repair. This process, largely driven by hypoxia-inducible factor, acts as a key regulator in promoting angiogenesis and vessel formation by triggering VEGF expression [56]. Exposure of SD rat extraction sockets to intermittent hypobaric hypoxia accelerated socket healing, evidenced by increased HIF-1α and VEGF mRNA expression and angiogenesis in hypoxic animals compared to the control groups [9]. Extraction sites filled with HIF-1α demonstrated an upregulation of VEGF, signifying increased angiogenesis in the healing tooth sockets. Further, osteoblast-derived VEGF stimulates osteogenic differentiation during bone development [56]. A similar study conducted by Lim et al. showed an increase in the number of blood vessels and new bone formation in the extraction sockets of beagle dogs [57].

Healing of the extraction socket is influenced by a variety of local, systemic, iatrogenic, and environmental factors of which vascularity is the most essential factor for osteogenesis [58]. Insufficient vascularity compromises bone healing by interrupting the nutritional and metabolic supply [8]. Type-H vessels support bone development by transporting oxygen, nutrients, growth factors, and osteoprogenitor cells to developing bone sites, thereby facilitating bone growth, and promoting bone formation. Type-H vessels, in contrast to type-L vessels, are linked to osteoprogenitor cells that express Osterix and have high expression levels of pro-osteogenic factors, such as bone morphogenetic proteins (BMPs), fibroblast growth factors (FGFs), and platelet-derived growth factors (PDGFs). Type-H vessels play a pivotal role in alveolar bone remodeling [59]. Hypoxia stabilizes HIF-1α in endothelial cells, which boosts the number of type-H vessels and Osterix-expressing cells, resulting in an increase in trabecular bone mass [60]. Yan et al. demonstrated an increased type-H blood vessels in healing tooth sockets [61]. The effect of hypoxia in inducing type-H vessels in alveolar socket healing needs further confirmation. Based on these reports, hypoxia-mediated HIF-1α activation stimulates both angiogenesis and osteogenesis, enabling it therapeutically valuable for improving the healing of extraction sockets and alveolar ridge preservation [55].

## 11 Therapeutic implications of hypoxia on alveolar bone remodeling

Alveolar bone health is important for both the retention and restoration of dentition. Alveolar bone loss is influenced by trauma, periodontal diseases, and periapical pathologies [62]. Currently, hypoxia-based strategies are gaining interests in tissue regeneration. Recent therapeutic interventions have explored the potential of in vitro and in vivo hypoxic stimulation in alveolar bone repair [9,26]. Gene therapies and pharmacological agents that activate and over express HIF-1α have been widely tested in alveolar bone regeneration [63,64]. Hypoxia mimetic agents (HMA) prevents HIF-1α degradation by inhibiting the PHD. Dimethyloxalylglycine (DMOG), a potential HIF-1α activator, inhibited alveolar bone loss in mouse models [63]. The effectiveness of such targeted immunomodulatory therapies for preventing alveolar bone resorption should be comprehensively investigated in human subjects. Tissue engineering approaches such as gene therapy that overexpresses HIF-1α, promotes alveolar bone defect osteogenesis. This mode is anticipated to provide an extended period of action compared to other local modes of HIF-1α activation [64]. One of the promising approaches to restore and regenerate bone is stem cell transplantation. Repairing bone defects in the elderly is a significant challenge, primarily because aging favors adipocytic differentiation over osteoblastic differentiation of BMSCs. Moreover, BMSCs cultured under normoxic conditions often struggle to survive in the hypoxic environments typically found in bone defects. The current strategy of hypoxic preconditioning of BMSCs improves their viability, promotes osteogenic differentiation, and significantly enhances the repair of mandibular bone defects in aged rats [65]. Furthermore, PDL stem cells cultured under severe hypoxic conditions (1 % O_2_) were capable of upregulating VEGF [66]. These findings pave the way for future periodontal research that can utilize hypoxic preconditioned cell-based therapies to potentially improve post-surgical alveolar bone healing in the elderly population. Adequate alveolar ridge dimension is a prerequisite for the stability of prosthetic replacements. Since hypoxia stimulates both angiogenesis and osteogenesis in extraction sites, leveraging hypoxic conditioning or HIF-1α activation could be an effective therapeutic strategy to accelerate socket healing and alveolar ridge preservation in the forthcoming years, ultimately enabling the functional restoration of dentition.

## 12 Dual role of hypoxia in alveolar bone remodeling: Impact of type, duration and exposure

Hypoxia exerts a dual influence on bone remodeling, affecting both bone formation and resorption. The duration and timing of both hypoxia and subsequent re-oxygenation may be critical in determining hypoxia's impact on bone metabolism [67]. In animal models, short-term hypoxia promoted bone repair, while chronic hypoxia resulted in bone loss [7,9,11]. It can be proposed that long-term and short-term hypoxia differently affect bone physiology due to the distinct signaling pathways they activate [68]. Continuous hypoxia (CH) reduced BMD in the mandible [34]. Additionally, both long-term continuous and intermittent hypoxia induce varied effects on bone remodeling [7,11]. CIH hypoxia exerts more deleterious effects on the alveolar bone compared to CCH. These findings imply that the reoxygenation periods in intermittent hypoxia (IH) worsen alveolar bone health.

In vitro studies typically employ short-term hypoxia for cell cultures. The outcomes of short-term continuous hypoxia on osteogenic differentiation of stem cells have been mixed. One study revealed increased osteogenic differentiation and enhanced alveolar bone defect repair [26], while other studies observed reduced osteogenic differentiation favoring of bone resorption [34,41]. However, the method of hypoxia induction and the type of stem cells used varied between these studies. The former study employed chemical induction with CoCl₂ in hPDLSCs, whereas the latter used a hypoxic chamber with 5 % O_2_ in BMSCs. This discrepancy suggests that cellular responses to hypoxia may vary depending on the method of induction, whether physiological or chemical.

The effects of hypoxia on alveolar bone are also influenced by the specific hypoxic model used. For instance, in vivo studies inducing short-term intermittent hypoxia have shown an enhancement in BMD and faster socket healing [9]. Conversely, in vitro studies demonstrated that short-term hypoxia followed by re-oxygenation in PDL cells can stimulate factors that promote bone resorption [38].

Hypoxia treatment appears to enhance the osteogenic activity of hPDLSCs primarily during the initial phase of exposure [26]. This indicates that the osteogenic effects of hypoxia on alveolar bone remodeling are time-dependent, with the beneficial impact of hypoxia diminishing as exposure lengthens. This decline in osteogenic potential during prolonged hypoxia may be attributed to the accumulation of ROS and acidic byproducts, which can eventually hinder bone remodeling [3].

The studies discussed in this review present conflicting findings on the role of hypoxia-mediated inflammatory pathways in alveolar bone remodeling. On one hand, hypoxia-induced upregulation of HIF-1α has been found to enhance inflammatory mediators, leading to increased alveolar bone resorption [7,40]. On the other hand, direct vector-mediated activation of HIF-1 resulted in the downregulation of pro-inflammatory mediators and a reduction in alveolar bone loss, suggesting that distinct molecular mechanisms are triggered by different stimuli [31]. These reports suggest that hypoxia triggers the expression of various cytokines in different cell types; however, the specific mechanisms underlying the induction of each cytokine remain a topic of debate. In the context of alveolar bone remodeling, hypoxia acts as a double-edged sword. While periodontal hypoxia in periodontitis contributes to alveolar bone resorption [21] before tooth extraction, induced hypoxic conditions can facilitate new bone formation after extraction [9]. Due to the intricate nature of hypoxia's impact on bone metabolism, a comprehensive understanding of its effects on alveolar bone under various conditions remains elusive. Further research is essential to elucidate the specific influences of different types, methods, and durations of hypoxia on alveolar bone remodeling.

## 13 Authors’ perspectives and recommendations

Hypoxia research began as early as 1945 and gained momentum with the discovery of HIF in 1995. From 2006 to 2016, hypoxia studies in dentistry, particularly concerning bone, expanded significantly. Early studies focused on hypoxia's effects on tissues, while recent research explores its therapeutic potential. Interest in hypoxia has surged anew following the systemic hypoxia observed during the COVID-19 pandemic [[24], [32]]. Our recent research using mouse models has shown that chronic, continuous hypoxia significantly and adversely affects oral tissues (unpublished data). Conducting hypoxia-based research presents several key challenges for researchers. A primary challenge is selecting the appropriate type and duration of hypoxia, especially in studies focused on the alveolar bone and periodontium. Researchers must align these parameters carefully with the study's objectives—whether they aim to assess hypoxia's impact on specific tissues or to explore its potential therapeutic benefits. Additionally, while animal models are invaluable for predicting human responses to hypoxic stress, they do not fully replicate human physiology, which limits the direct translation of findings. In vitro and animal studies have been essential for understanding hypoxia's pathogenesis, effects, and possible treatments, though their clinical application remains restricted. Another major challenge is that many hypoxia models induce generalized hypoxia, underscoring the need to develop more localized models that better represent the conditions seen in dental diseases. Furthermore, exploring genetic approaches to induce hypoxia for oral tissue regeneration is particularly promising, though complex; these methods may offer significant advantages over traditional pharmacologically induced hypoxia, yet they require further refinement for effective utilization.

## 14 Conclusion

Oxygen is essential for alveolar bone growth, remodeling, and repair. Hypoxia, in all its forms, has a significant impact on alveolar bone health, primarily through the activation of HIF. Short-term hypoxia appears to be more beneficial for alveolar bone than long-term exposure, whereas CIH is more detrimental to alveolar bone health than CCH. The mode, degree, and duration of hypoxia trigger distinct regulatory mechanisms, leading to varied responses in alveolar bone remodeling. However, critical gaps remain in our understanding of how these factors influence bone metabolism. Future research should focus on elucidating the time- and dose-dependent cellular responses to both continuous and intermittent hypoxic stimuli. A clearer understanding of these factors is crucial to effectively modulate hypoxia in a therapeutic context. Additionally, there is a noticeable lack of experimental research on humans to explore how alveolar bone responds to different hypoxic stimuli. Given that HIF activation has been shown to enhance extraction socket healing, there is an urgent need for clinical trials to evaluate therapeutic strategies that utilize hypoxia to promote alveolar bone regeneration.

## CRediT authorship contribution statement

**Sangeetha Narasimhan:** Writing – original draft, Supervision, Conceptualization. **Sausan Al Kawas:** Writing – review & editing, Supervision. **Shishir Ram Shetty:** Validation, Data curation. **Hiba Saad Al-Daghestani:** Resources, Data curation. **Rani Samsudin:** Writing – review & editing, Conceptualization.

## Availability of data and materials

No data was used for the research described in the article.

## Funding sources

None to Declare.

## Declaration of competing interest

The authors declare that they have no known competing financial interests or personal relationships that could have appeared to influence the work reported in this paper.
